# Sex-based differences in veterans with pulmonary hypertension: Results from the veterans affairs-clinical assessment reporting and tracking database

**DOI:** 10.1371/journal.pone.0187734

**Published:** 2017-11-09

**Authors:** Corey E. Ventetuolo, Edward Hess, Eric D. Austin, Anna E. Barón, James R. Klinger, Tim Lahm, Thomas M. Maddox, Mary E. Plomondon, Lauren Thompson, Roham T. Zamanian, Gaurav Choudhary, Bradley A. Maron

**Affiliations:** 1 Department of Medicine, Alpert Medical School of Brown University, Providence, Rhode Island, United States of America; 2 Department of Health Services, Policy and Practice, Brown University School of Public Health, Providence, Rhode Island, United States of America; 3 Veterans Affairs Eastern Colorado Health Care System, Denver, Colorado, United States of America; 4 Division of Pediatric Pulmonary, Allergy, and Immunology, Vanderbilt University, Nashville, Tennessee, United States of America; 5 Division of Pulmonary, Critical Care, Occupational and Sleep Medicine, Indiana University School of Medicine, Indianapolis, Indiana, United States of America; 6 Richard L. Roudebush Veterans Affairs Medical Center, Indianapolis, Indiana, United States of America; 7 University of Colorado School of Medicine, Aurora, Colorado, United States of America; 8 Vera Moulton Wall Center for Pulmonary Vascular Disease, Stanford University School of Medicine, Stanford, California, United States of America; 9 Providence Veterans Affairs Medical Center, Providence, Rhode Island, United States of America; 10 Boston Veterans Affairs Healthcare System, Boston, Massachusetts, United States of America; 11 Brigham and Women’s Hospital and Harvard Medical School, Boston, Massachusetts, United States of America; University of Alabama at Birmingham, UNITED STATES

## Abstract

Women have an increased risk of pulmonary hypertension (PH) but better survival compared to men. Few studies have explored sex-based differences in population-based cohorts with PH. We sought to determine whether sex was associated with hemodynamics and survival in US veterans with PH (mean pulmonary artery pressure [mPAP] ≥ 25 mm Hg) from the Veterans Affairs Clinical Assessment, Reporting, and Tracking database. The relationship between sex and hemodynamics was assessed with multivariable linear mixed modeling. Cox proportional hazards models were used to compare survival by sex for those with PH and precapillary PH (mPAP ≥ 25 mm Hg, pulmonary artery wedge pressure [PAWP] ≤ 15 mm Hg and pulmonary vascular resistance [PVR] > 3 Wood units) respectively. The study population included 15,464 veterans with PH, 516 (3%) of whom were women; 1,942 patients (13%) had precapillary PH, of whom 120 (6%) were women. Among those with PH, women had higher PVR and pulmonary artery pulse pressure, and lower right atrial pressure and PAWP (all p <0.001) compared with men. There were no significant differences in hemodynamics according to sex in veterans with precapillary PH. Women with PH had 18% greater survival compared to men with PH (adjusted HR 0.82, 95% CI 0.69–0.97, p = 0.020). Similarly, women with precapillary PH were 29% more likely to survive as compared to men with PH (adjusted HR 0.71, 95% CI 0.52–0.98, p = 0.040). In conclusion, female veterans with PH have better survival than males despite higher pulmonary afterload.

## Introduction

Pulmonary hypertension (PH) refers to an elevation in pulmonary artery pressures (PAP) due to various etiologies. Precapillary PH is characterized by an increase in pulmonary vascular resistance (PVR) which may be idiopathic, heritable, or associated with systemic diseases (all referred to as World Health Organization [WHO] Group 1 pulmonary arterial hypertension [PAH]) [1]. As many as 80% of prevalent WHO Group 1 patients are women [2]. Precapillary PH also occurs in chronic lung disease and sleep disordered breathing (WHO Group 3 PH), chronic thromboembolic disease (WHO Group 4 PH), or combined with post-capillary PH in left heart disease (WHO Group 2 PH) [3]. PH is a frequent complication of highly prevalent cardiopulmonary diseases and is associated with increased morbidity and mortality when present [4–9]. In the last two decades, disease burden for all types of PH has increased, particularly among women [4]. Despite greater disease prevalence, it appears that women with PH have improved survival compared to men with PH [5, 10–13], but sex-based differences in outcomes have not been well-characterized outside of WHO Group 1 PAH.

Hemodynamics are required for the diagnosis and treatment of PH (i.e., to detect increased PVR and distinguish pre- from post-capillary PH) and are established predictors of survival [14–17]. We have previously shown that in patient-level data from clinical trials men with PAH had more severe hemodynamic abnormalities as compared to women with PAH [18]. Similarly, in the Registry to Evaluate Early and Long-term PAH Disease Management (REVEAL) men with PAH had more hemodynamic derangement than women with PAH [14]. Age appeared to modify the associations between sex and hemodynamics in our prior study and sex and survival in REVEAL, with some discrepancies observed between the two studies [14,18]. Since both were limited to patients with WHO Group 1 PAH, it is unknown whether these observations extend to other quite common forms of PH. Large epidemiologic cohorts with hemodynamic assessments in which to replicate these observations in a “real world” unselected population of men and women with all forms of PH have to this point been lacking.

Veterans are characterized by a particularly high prevalence of PH, and the presence of PH is a strong predictor of increased mortality in this population [8]. We therefore sought to determine whether sex is associated with hemodynamics and survival in a cohort of US veterans who underwent right heart catheterization (RHC) in whom outcomes were tracked as part of a national clinical quality initiative, the Veterans Affairs (VA) Clinical Assessment, Reporting, and Tracking (CART) program [19]. We hypothesized that in PH and precapillary PH, women would have less severe hemodynamic impairment as compared to men. Second, we hypothesized that women would have more favorable survival as compared to men with PH and precapillary PH respectively. Finally, we explored whether survival differences (if present) could be explained by hemodynamic differences and whether age modified any of the observed relationships.

## Methods

### Study population

We evaluated all veterans with procedural data recorded in CART who underwent RHC as an outpatient or inpatient in the VA system between October 1, 2007 and September 30, 2013. In patients undergoing multiple RHCs, the first RHC was used to characterize hemodynamics and values from this index procedure were included in the analysis. Further details about the 76 participating centers and RHC volume by center have previously been published [8,19]. Catheterization reports generated by the CART system pass rigorous quality control [20], in concordance with the definitions and standards of the American College of Cardiology’s National Cardiovascular Data Registry [21].

The source population included all subjects with RHC data available including values for mean pulmonary artery pressure (mPAP), pulmonary artery wedge pressure (PAWP), cardiac output (CO) measured by either assumed Fick and/or thermodilution methods, and height and weight for calculation of body surface area. Variables were assessed for physiologic plausibility and validated by two cardiologists with expertise in hemodynamic analysis and were assigned a corrected value based on ancillary data (i.e., values that could be confirmed in the medical record or could be calculated from other variables), or a value of missing where correct value(s) could not be identified, as previously described [8]. PVR was calculated as (mPAP—PAWP)/CO and pulmonary artery pulse pressure (PAPP), a marker of RV loading that predicts poor outcomes in various forms of PH [22,23], was calculated as systolic PAP—diastolic PAP.

The final study population included those with PH (defined as a mPAP ≥ 25 mm Hg) and those with precapillary PH (defined as a mPAP ≥ 25 mm Hg, a PAWP ≤ 15 mm Hg, and a PVR > 3 Wood units) [24] stratified by sex.

### Covariates

Clinical characteristics representing a comprehensive group of risk factors for the development of PH [24–26] were captured via CART or from VA administrative data [8, 27–29]. Covariates included age, sex, race (black, white, or other which included American Indian, Alaskan Native, Asian, Native Hawaiian, or other Pacific Islander), body mass index (BMI) (categorized as underweight, overweight, obese, and severely obese), history of systemic hypertension, left heart failure (i.e., heart failure due to left ventricular systolic or diastolic dysfunction), congestive heart failure (i.e., heart failure that may include RV or biventricular dysfunction), diabetes mellitus, coronary artery disease (CAD) (considered present if history of myocardial infarction, percutaneous coronary intervention, coronary artery bypass graft surgery, or presence of any obstructive disease on cardiac catheterization was present), chronic obstructive pulmonary disease (COPD), human immunodeficiency virus (HIV), liver cirrhosis (viral and non-viral hepatitis), chronic kidney disease (including patients receiving renal replacement therapy), connective tissue disease, atrial fibrillation or flutter, interstitial lung disease, obstructive sleep apnea (OSA) and/or sleep disordered breathing, pulmonary embolism, any valvular disease (regurgitation or stenosis), and tobacco use. Depression and posttraumatic stress disorder (PTSD) were also included as their prevalence varies by sex and they are associated with cardiovascular outcomes and survival in US veterans and other populations [27, 28].

### Outcomes

Our primary outcome of interest was survival. A minimum of one year of follow-up survival data was available for all subjects. CART data was combined with longitudinal records captured in the VA electronic health record including survival and also linked to claims data outside of the VA system. The Colorado Multiple Institutional Review Board approved this study.

### Statistical analysis

Continuous data were summarized as median (interquartile range [IQR]) and categorical data was summarized as percentages (frequency) with Kruskal-Wallis or Chi-Square tests for continuous or categorical comparisons respectively. The relationship between sex and hemodynamic outcomes in the two cohorts with PH and precapillary PH was assessed with multivariable linear mixed modeling and expressed as least square means. Cox proportional hazards models were used to examine the relationship between sex and survival in the PH and precapillary PH cohorts. Kaplan Meier plots were constructed stratified by sex for both groups.

All linear mixed models included a random intercept for hospital site and were adjusted for multiple covariates of interest (detailed above) including: age, sex, race, BMI, history of systemic hypertension, left heart failure and/or congestive heart failure, diabetes mellitus, CAD, COPD, HIV, liver disease, chronic kidney disease/dialysis, connective tissue disease, atrial fibrillation or flutter, interstitial lung disease, OSA and/or sleep disordered breathing, pulmonary embolism, any valvular regurgitation or stenosis, tobacco use, cancer, depression, PTSD, and inpatient status. Cox proportional hazards models included the same covariates for the PH cohort. A low number of events for women in the precapillary PH group only allowed simultaneous adjustment for a much smaller number of covariates (relative to the PH cohort). Age, sex, race, BMI, and inpatient status were automatically included in Cox models for the precapillary PH group. Potential confounding by the remaining covariates was assessed by adding each individually to a model containing the five aforementioned covariates to assess the impact each had on the effect estimate for sex. Chronic kidney disease/dialysis was included in the final Cox models as it had the greatest impact (changing the parameter estimate for sex by > 15%, whereas all others had a much smaller impact, with a change of typically a few percent in all cases).

Finally, exploratory analyses were conducted to determine whether 1) age modified the sex-hemodynamic and sex-survival relationships and 2) whether a significant proportion of the relationship between sex and survival was mediated (explained by) by the hemodynamic variables. We assessed for a sex by age interaction and included an interaction term (sex*age) in the multivariable models when there was evidence of effect modification (p < 0.05) to determine whether there was a change in effect estimates. Age was assessed as both a categorical variable (age < 55, [55–65], and ≥ 65) and a continuous variable.

Modeling for the mediator analysis required that 1) multivariate linear mixed models confirmed that sex was an independent predictor of hemodynamics and 2) Cox proportional hazards modeling demonstrated sex was a significant predictor of survival after adjustment for multiple covariates. A test statistic for mediation was then created using the product method [30,31], such that “a” was the coefficient of sex in a multivariate mixed model with the hemodynamic mediator of interest as the outcome and “b” was the coefficient of the hemodynamic mediator in the adjusted Cox model for survival:

Zstatistic=numerator÷denominator,

where

Numerator=(a÷standarderrorofa)×(b÷standarderrorofb)

$$\text{Denominator}\;=\;\sqrt{[a^{2}÷{\left(standard\;error\;of\;b\right)}^{2}+\;b^{2}÷\;{\left(standard\;error\;of\;b\right)}^{2}+1}]$$

The Z statistic was then compared to the percentiles of a standard normal distribution, and mediation was considered significant if p< 0.05.

Each hemodynamic variable was treated as a separate hypothesis, and therefore modeled independently in all models. Data preparation and analyses were conducted using SAS version 9.4 (SAS Institute, Cary North Carolina) and R version 3.3.1. Statistical significance was defined as p<0.05.

## Results

We identified 28,775 index RHC records available for analysis in which subjects had mPAP ≥ 25 mm Hg. Of these, N = 15,464 (54%) had CO recorded (by Fick, thermodilution, or both). The final dataset included 22 subjects (0.1%) who were assigned a corrected hemodynamic measurement based on ancillary data (i.e., values that could be confirmed in the medical record or could be calculated from other variables). The final study population included 15,464 subjects with PH, 516 (3%) of whom were women. There were 1,942 (13%) subjects with precapillary PH, 120 (6%) of whom were women.

Characteristics of the two study cohorts are presented in Tables 1 and 2, respectively, by sex. Among those with PH, women tended to be younger, black, more severely obese (BMI ≥ 33.5 kg/m^2^) and were less likely to have systemic hypertension as compared to men (Table 1). While these differences were highly statistically significant due to the large sample size, the relative proportions were similar. As expected, men were much more likely to have a diagnosis of left heart failure, CAD, atrial fibrillation and chronic kidney disease while women were much more likely to have a diagnosis of depression. There were similar rates of pulmonary comorbidities and PTSD, while men were more likely to have been inpatients during cardiac catheterization and have cancer. In those with precapillary PH, these observations were similar (Table 2). Women with precapillary PH were more likely to have a history of connective tissue disease than men with precapillary PH (13.3% vs. 4.4%, p < 0.001).

**Table 1:** Characteristics of the total cohort with pulmonary hypertension*, by sex.

Variable	All	Women	Men	P value
Number	15464	516	14948	
Age, years	65.2 (60.4,73.3)	59.3 (53.3,66.9)	65.3 (60.6,73.5)	<0.001
Race, % (n)				<0.001
White	73.3 (11333)	60.7 (313)	73.7 (11020)	
Black	19.6 (3038)	31.6 (163)	19.2 (2875)	
Other	7.1 (1093)	7.8 (40)	7.0 (1053)	
Body mass index, % (n)				<0.001
Normal (18.5–25 kg/m^2^)	17.6 (2725)	17.4 (90)	17.6 (2635)	
Underweight (<18.5 kg/m^2^)	0.9 (133)	2.1 (11)	0.8 (122)	
Overweight (25–30 kg/m^2^)	29.5 (4561)	23.6 (122)	29.7 (4439)	
Obese (30–35 kg/m^2^)	24.7 (3814)	23.8 (123)	24.7 (3691)	
Severely obese (≥35 kg/m^2^)	27.4 (4231)	32.9 (170)	27.2 (4061)	
Systemic hypertension, % (n)	89.9 (13901)	82.9 (428)	90.1 (13473)	<0.001
Diabetes mellitus, % (n)	52.6 (8131)	44.8 (231)	52.8 (7900)	<0.001
Tobacco use, % (n)	59.3 (9171)	47.9 (247)	59.7 (8924)	<0.001
Cardiac conditions				
Congestive heart failure, % (n)	69.4 (10728)	56.2 (290)	69.8 (10438)	<0.001
Left heart failure, % (n)	15.2 (2347)	9.1 (47)	15.4 (2300)	<0.001
Coronary artery disease, % (n)	57.9 (8957)	30.4 (157)	58.9 (8800)	<0.001
Valvular disease, % (n)	39.4 (6086)	37.6 (194)	39.4 (5892)	0.432
Atrial fibrillation or flutter, % (n)	33.3 (5145)	19.6 (101)	33.7 (5044)	<0.001
Pulmonary conditions				
COPD, % (n)	38.0 (5882)	32.9 (170)	38.2 (5712)	0.017
Obstructive sleep apnea, % (n)	13.3 (2062)	12.4 (64)	13.4 (1998)	0.571
Pulmonary embolism, % (n)	3.9 (602)	5.4 (28)	3.8 (574)	0.086
Systemic conditions				
Chronic kidney disease, % (n)	35.2 (5442)	18.2 (94)	35.8 (5348)	<0.001
HIV infection, % (n)	0.5 (79)	0.2 (1)	0.5 (78)	0.476
Liver cirrhosis, % (n)	7.4 (1139)	5.8 (30)	7.4 (1109)	0.198
Connective tissue disease, % (n)	3.0 (465)	10.3 (53)	2.8 (412)	<0.001
Sickle cell anemia, % (n)	0.0 (6)	0.2 (1)	0.0 (5)	0.495
Posttraumatic stress disorder, % (n)	13.5 (2082)	13.2 (68)	13.5 (2014)	0.899
Depression, % (n)	27.9 (4311)	44.2 (228)	27.3 (4083)	<0.001
Inpatient status, % (n)	52.1 (8063)	43.4 (224)	52.4 (7839)	<0.001
Active cancer, % (n)	16.2 (2504)	11.4 (59)	16.4 (2445)	0.003

*Defined as a mean pulmonary artery pressure ≥ 25 mm Hg. Data are shown as % (n) or median (interquartile range). COPD = chronic obstructive pulmonary disease; HIV = human immunodeficiency virus

**Table 2:** Characteristics of the subgroup with precapillary pulmonary hypertension†, by sex.

Variable	All	Women	Men	P value
Number	1942	120	1822	
Age, years	65.7 (60.5,74.3)	57.6 (52.3,67.3)	66.1 (61.1,74.6)	<0.001
Race, % (n)				<0.001
White	68.3 (1319)	51.7 (62)	69.8 (1271)	
Black	24.1 (466)	38.3 (46)	23.1 (420)	
Other	7.5 (145)	10.0 (12)	7.2 (131)	
Body mass index, % (n)				0.399
Normal (18.5–25 kg/m^2^)	28.4 (551)	24.2 (29)	28.6 (522)	
Underweight (<18.5 kg/m^2^)	2.1 (41)	1.7 (2)	2.1 (39)	
Overweight (25–30 kg/m^2^)	32.5 (632)	30.8 (37)	32.7 (595)	
Obese (30–35 kg/m^2^)	21.0 (408)	21.7 (26)	21.0 (382)	
Severely obese (≥35 kg/m^2^)	16.0 (310)	21.7 (26)	15.6 (284)	
Systemic hypertension, % (n)	86.3 (1675)	85.8 (103)	86.3 (1572)	0.753
Diabetes mellitus, % (n)	44.8 (870)	42.5 (51)	45.0 (819)	0.424
Tobacco use, % (n)	64.4 (1251)	48.3 (58)	65.5 (1193)	<0.001
Cardiac conditions				
Congestive heart failure, % (n)	59.5 (1156)	45.0 (54)	60.5 (1102)	0.001
Left heart failure, % (n)	12.1 (235)	1.7 (2)	12.8 (233)	0.001
Coronary artery disease, % (n)	49.6 (963)	26.7 (32)	51.1 (931)	<0.001
Valvular disease, % (n)	27.0 (525)	19.2 (23)	27.6 (502)	0.058
Atrial fibrillation or flutter, % (n)	24.7 (479)	9.2 (11)	25.7 (468)	<0.001
Pulmonary conditions				
COPD, % (n)	54.4 (1057)	45.0 (54)	55.0 (1003)	0.041
Obstructive sleep apnea, % (n)	11.2 (218)	10.0 (12)	11.3 (206)	0.772
Pulmonary embolism, % (n)	4.8 (93)	4.2 (5)	4.8 (88)	0.913
Systemic conditions				
Chronic kidney disease, % (n)	29.4 (571)	14.2 (17)	30.4 (554)	<0.001
HIV infection, % (n)	0.7 (14)	0.8 (1)	0.7 (13)	1.000
Liver cirrhosis, % (n)	9.7 (188)	5.8 (7)	9.9 (181)	0.189
Connective tissue disease, % (n)	4.9 (96)	13.3 (16)	4.4 (80)	<0.001
Sickle cell anemia, % (n)	0.2 (3)	0.8 (1)	0.1 (2)	0.453
Posttraumatic stress disorder, % (n)	12.1 (235)	14.2 (17)	12.0 (218)	0.567
Depression, % (n)	26.1 (506)	46.7 (56)	24.7 (450)	<0.001
Inpatient status, % (n)	43.0 (835)	29.2 (35)	43.9 (800)	0.002
Active cancer, % (n)	16.4 (319)	9.2 (11)	16.9 (308)	0.037

^†^Defined as a mean pulmonary artery pressure ≥ 25 mm Hg, a pulmonary capillary wedge pressure ≤ 15 mm Hg, and a pulmonary vascular resistance > 3 Wood units. Data are shown as % (n) or median (interquartile range). COPD = chronic obstructive pulmonary disease; HIV = human immunodeficiency virus

Unadjusted median (IQR) hemodynamic values by sex are presented in Table 3 for the cohorts. There were moderate elevations in pulmonary pressures across all groups. Women with PH had higher PVR and PAPP and lower PAWP and right atrial pressure (RAP) (p < 0.001 for all) compared to men (Table 4). For example, women with PH tended to have approximately a 1 mm Hg lower RAP (-0.9 mm difference, 95% confidence interval [CI] -1.4 to -0.4 mm Hg, p < 0.001) and a 2 mm Hg higher PAPP (1.9 mm Hg difference, 95% CI 0.9 to 2.8, p < 0.001) as compared to men with PH controlling for demographics, body size, medical comorbidities, inpatient status, and site. In the cohort with precapillary PH, there were no significant differences in hemodynamics although effect estimates and trends were similar (higher PVR and PAPP, lower PAWP and RAP in women compared to men) in this smaller sample. Despite higher levels of pulmonary afterload (PVR and PAPP) in women with precapillary PH, there were no differences in RV diastolic pressures between men and women (p = 0.78) with precapillary PH.

**Table 3:** Unadjusted hemodynamic variables for pulmonary hypertension cohort and precapillary pulmonary hypertension subgroup, by sex.

	Pulmonary hypertension*					Precapillary pulmonary hypertension†				
Variable	N missing	All	Women	Men	P value	N missing	All	Women	Men	P value
		15464	516	14948			1942	120	1822	
RAP, mm Hg	143	12.0 (8.0,15.0)	10.0 (7.0,14.0)	12.0 (8.0,15.0)	<0.001	10	8.0 (5.0, 10.0)	8.0 (5.0, 10.0)	8.0 (5.0, 10.0)	0.908
mPAP, mm Hg	0	34.0 (29.0,41.0)	33.0 (28.8,40.0)	34.0 (29.0,41.0)	0.731	0	32.0 (28.0, 40.0)	32.0 (28.0, 41.0)	32.0 (28.0, 40.0)	0.907
PAWP, mm Hg	0	21.0 (16.0,26.0)	19.0 (14.0,25.0)	21.0 (16.0,26.0)	<0.001	0	12.0 (9.0,14.0)	11.0 (9.0,14.0)	12.0 (9.0,14.0)	0.555
CI, L/min/m^2^	0	2.3 (1.9,2.8)	2.4 (2.0,2.9)	2.3 (1.9,2.8)	0.005	0	2.2 (1.9,2.6)	2.4 (2.0,2.8)	2.2 (1.9,2.6)	0.008
PVR, Wood units	46	2.5 (1.7,3.8)	3.1 (2.0,4.6)	2.5 (1.7,3.7)	<0.001	0	4.6 (3.6,6.5)	4.9 (3.8,7.0)	4.5 (3.6,6.5)	0.189
PAPP, mm Hg	34	28.0 (21.0,35.0)	27.0 (22.0,35.8)	28.0 (21.0,35.0)	0.448	8	31.0 (24.0,40.0)	31.0 (23.0,43.0)	31.0 (24.0,40.0)	0.575

* Defined as a mean pulmonary artery pressure (mPAP) ≥ 25 mm Hg;

^†^Defined as a mPAP ≥ 25 mm Hg, a pulmonary artery wedge pressure (PAWP) ≤ 15 mm Hg, and a pulmonary vascular resistance (PVR) > 3 Wood units; RAP = right atrial pressure; CI = cardiac index; PAPP = pulmonary artery pulse pressure.

**Table 4:** Associations between sex and hemodynamics in pulmonary hypertension* cohort and precapillary pulmonary hypertension subgroup after multivariable adjustment‡.

	Pulmonary hypertension*				Precapillary pulmonary hypertension†			
Variable	Women	Men	Difference, women vs. men	P value	Women	Men	Difference, women vs. men	P value
RAP, mm Hg	12.3 (11.6,12.9)	13.2 (12.7,13.7)	-0.9 (-1.4,-0.4)	<0.001	8.1 (7.0,9.2)	8.2 (7.4,9.0)	-0.2 (-1.0,0.7)	0.710
mPAP, mm Hg	36.3 (35.4,37.3)	36.0 (35.3,36.7)	0.4 (-0.4,1.1)	0.352	35.9 (33.7,38.0)	36.7 (35.1,38.3)	-0.8 (-2.5,0.8)	0.318
PAWP, mm Hg	19.1 (18.3,20.0)	20.6 (20.0,21.3)	-1.5 (-2.1,-0.9)	<0.0001	10.8 (10.1,11.6)	10.5 (9.9,11.1)	0.3 (-0.3,0.9)	0.278
CI, L/min/m^2^	2.6 (2.5,2.7)	2.6 (2.5,2.6)	0.0 (0.0,0.1)	0.132	2.5 (2.3,2.6)	2.4 (2.3,2.5)	0.1 (0.0,0.2)	0.154
PVR, Wood units	4.2 (4.0,4.5)	3.4 (3.2,3.6)	0.8 (0.6,1.0)	<0.0001	6.1 (5.4,6.8)	5.8 (5.3,6.3)	0.3 (-0.3,0.8)	0.283
PAPP, mm Hg	32.0 (30.7,33.2)	30.1 (29.2,31.0)	1.9 (0.9,2.8)	0.0001	37.2 (34.1,40.3)	36.7 (34.5,39.0)	0.5 (-1.9,2.9)	0.700

Data are presented as least square means estimates (95% confidence interval).

* Defined as a mean pulmonary artery pressure (mPAP) ≥ 25 mm Hg;

^†^Defined as a mPAP ≥ 25 mm Hg, a pulmonary artery wedge pressure (PAWP) ≤ 15 mm Hg, and a pulmonary vascular resistance (PVR) > 3 Wood units; RAP = right atrial pressure; CI = cardiac index; PAPP = pulmonary artery pulse pressure.

^‡^ Models included a random intercept for hospital site and were adjusted for age, sex, race, body mass index, systemic hypertension, left heart failure and/or congestive heart failure, diabetes mellitus, coronary artery disease, chronic obstructive pulmonary disease, human immunodeficiency virus infection, liver disease, chronic kidney disease, connective tissue disease, atrial fibrillation or flutter, interstitial lung disease, obstructive sleep apnea and/or sleep disordered breathing, pulmonary embolism, any valvular disease, tobacco use, cancer, depression, posttraumatic stress disorder, and inpatient status.

**Fig 1:**
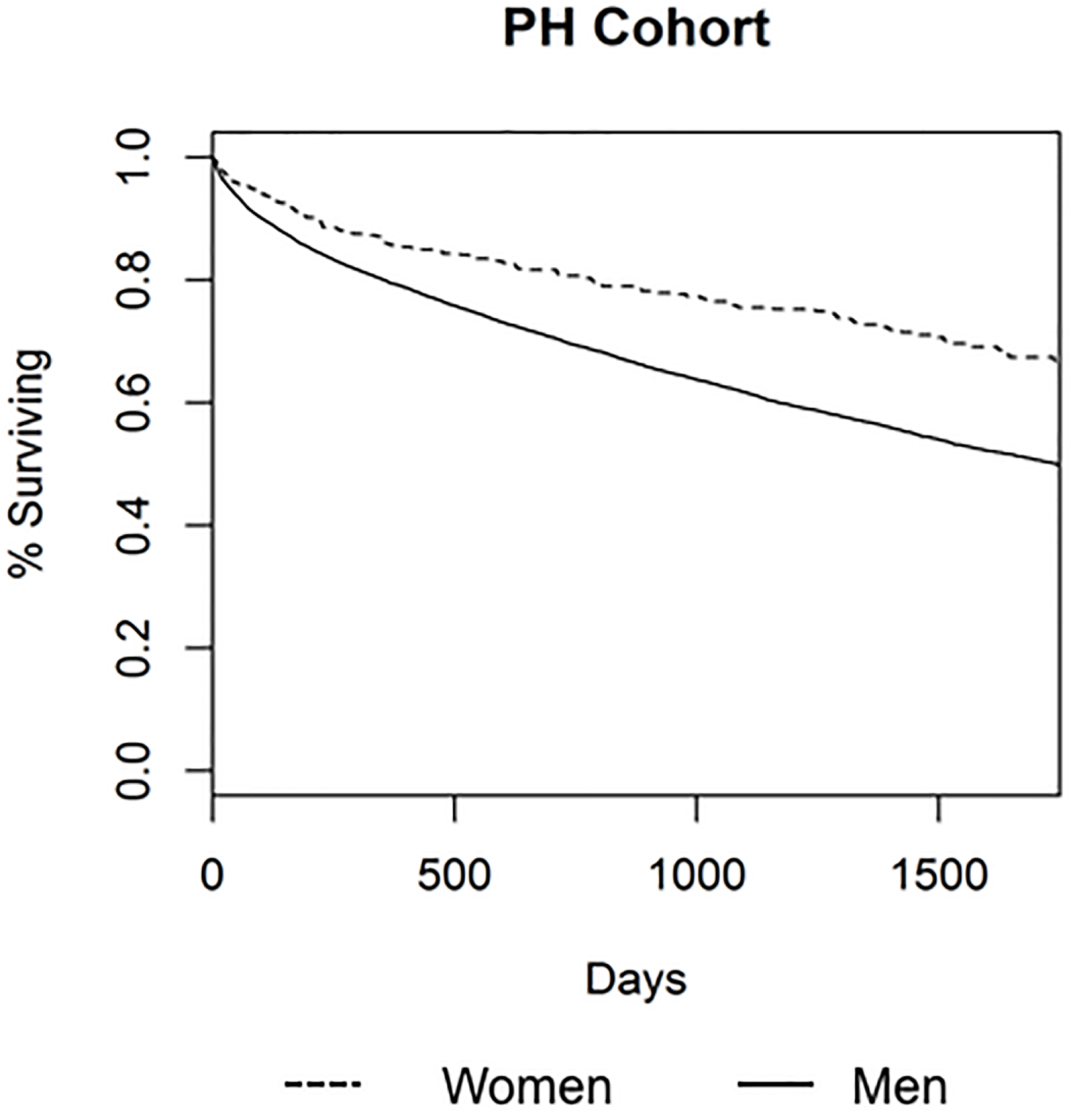
Kaplan-Meier curve for cohort of subjects with pulmonary hypertension (PH) by sex. Dotted line is women, solid line is men.

**Fig 2:**
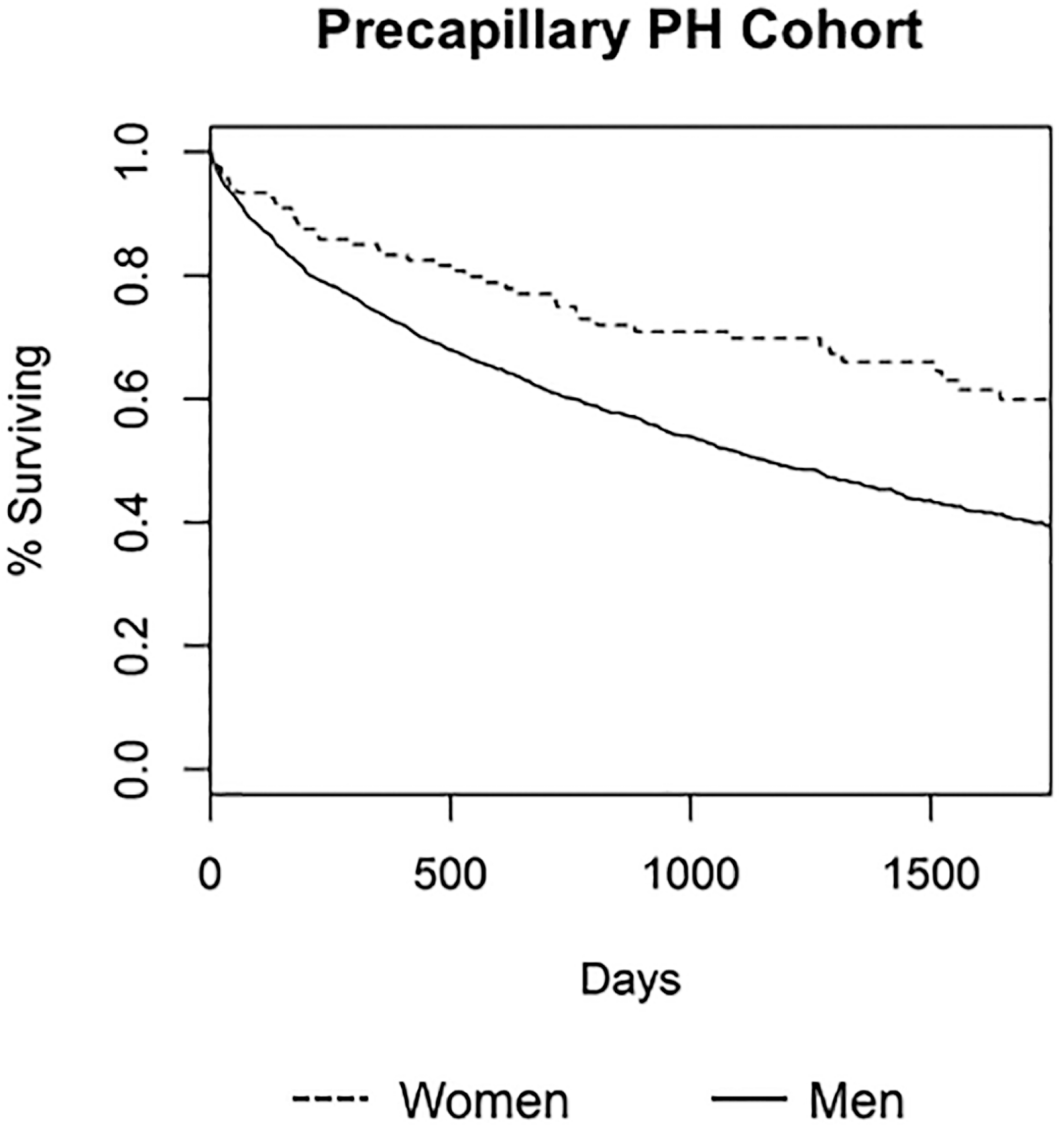
Kaplan-Meier curve for cohort of subjects with precapillary pulmonary hypertension (PH) by sex. Dotted line is women, solid line is men.

Women with PH had 18% increased survival compared to men with PH (hazard ratio [HR] 0.82, 95% CI 0.69–0.97, p = 0.020) after adjustment for multiple clinical variables and potential confounders. Similarly, in the precapillary PH subgroup women had a 29% reduction in the risk of death as compared to men although precision was decreased for this comparison due to a smaller sample size (HR 0.71, 95% CI 0.52–0.98, p = 0.040). Figs 1 and 2 demonstrate the Kaplan-Meier survival curves for PH and precapillary PH respectively for up to 1750 days after RHC.

After confirming that sex was significantly associated with both hemodynamics and survival in the PH cohort, we sought to determine if hemodynamics explained a significant proportion of the sex-survival relationship in those with PH. None of the hemodynamic parameters met the threshold for mediation (data not shown; p > 0.05 for all). In our exploratory analyses age did not modify the associations seen between sex and hemodynamics or sex and survival (data not shown).

## Discussion

In a large cohort of US veterans with PH women have different hemodynamic profiles and improved survival compared to men. Hemodynamics in women were characterized by a more compensatory RV response (lower RAP) despite increased afterload (higher PVR, higher PAPP) as compared to men; assessment for age-related interactions did not influence these results. Sex-based hemodynamic and survival differences persisted after adjustment for multiple comorbid conditions and potential confounders. In veterans with precapillary PH, survival differences persisted and hemodynamic patterns were similar, although not statistically significant. The difference in survival between women and men with PH was not mediated by the hemodynamic differences observed by sex. These results suggest distinct sex-based phenotypes in an unselected, population-based cohort of veterans with elevated pulmonary pressures.

Female sex is a well-known risk factor for WHO Group 1 PAH, but recent epidemiologic data suggests that men with PAH have poorer survival than women with PAH [4, 11, 12]. Hormonal regulation of cardiopulmonary function, sex-based differences in response to PAH therapy [32, 33], or differences in RV adaptation have been proposed as possible explanations for these observations. RV function has been identified as a major determinant of outcome in PAH, but also in PH due to left heart disease [5–7]. Heart failure with preserved ejection fraction (HFpEF) is more common in women [34, 35], yet women are less likely to develop RV dysfunction than men with HFpEF [13]. In idiopathic PAH, women have greater improvements in RV function with PAH therapy despite similar reductions in PVR as compared to men, and sex-based differences in the RV response explain a significant portion of the survival benefit seen in women as compared to men [36]. Women demonstrated lower RAP as compared to men in our study. Elevated RAP occurs in RV failure and has been consistently associated with poor survival in pulmonary vascular disease [15,16]. The associations demonstrated here are akin to earlier observations from “pure” WHO Group 1 PAH patients as well as patients with HFpEF and presumed pulmonary venous (or postcapillary) PH, suggesting persistent sex-based phenotypes across all types of pulmonary vascular disease.

The principal determinant of RV afterload is classically thought of as PVR, but more recently stiffening of large pulmonary arteries has also been shown to be important. Our findings that survival in women with PH was increased compared to men with PH despite higher PVR and higher PAPP (a measurement of RV loading) suggests that sex-specific RV adaptation patterns may exist and account for differences in survival. These data provide a clinical correlate to converging lines of experimental data showing a favorable effect by estrogen on pulmonary artery-RV coupling in PH *in vivo* [37–39]. In the systemic circulation, female sex and sex hormones have been proposed to contribute to vascular-ventricular coupling as well [40,41]. Assessment of RV function was limited to central hemodynamic measurements in our study and hemodynamic differences did not mediate the survival differences observed, perhaps due to sample or effect size limitations; more robust measures of RV morphology (e.g., echocardiography or cardiac magnetic resonance imaging) may have improved sensitivity to detect such relationships.

While our observations are certainly not generalizable to WHO Group 1 PAH, our findings may be more representative of a “real world” population of patients with PH. In fact, subgroups of classically idiopathic PAH, pre- and post-capillary PH patients may have similar genetic features and hemodynamic derangements that contribute to poor outcomes in all groups [3, 42]. These clinical phenotypes may represent a spectrum of pulmonary vascular abnormalities with shared pathobiologic mechanisms. As in right heart failure, women with left heart failure have a better prognosis, but this is not explained by the etiology of heart failure or degree of left ventricular dysfunction [43–46] (interestingly PAWP was lower in women than in men in our study). It is plausible that sex-based prevalence and outcome differences in left heart failure are driven by differences in pulmonary hemodynamics and the right heart and that sex and/or sex hormones may play an important role in PH regardless of how it is classified clinically.

We failed to detect any significant interactions between sex and age for the outcomes of hemodynamics or survival, which may be due to the low number of younger age subjects (particularly women) in this population. We have previously shown that in clinical trial participants with WHO Group 1 PAH, men had greater hemodynamic burden (including higher RAP, as seen here, but also higher mPAP and PVR) as compared to women, but some of these differences were attenuated after age 45 [18]. In REVEAL, men with PAH had higher RAP and mPAP at diagnosis (as well as worse survival especially in those older than 60 years of age) as compared to women with PAH [12–14]. These observations have not been consistent across all WHO Group 1 PAH registries [47,48], however, and it is therefore not surprising that our results from an unselected veteran population would be somewhat inconsistent with prior reports. Given the average age of the study subjects, it is likely that almost all women included were post-menopausal. Although we do not have confirmation of menopausal status, the inclusion of pre-menopausal women in the study sample were this data available may have provided additional insights.

There was a greater proportion of black women compared to men in both the PH and the precapillary PH groups. Observational studies have shown a more exaggerated female predominance among blacks as compared to whites with WHO Group 1 PAH [2,16]. Outcomes in both right and left heart failure may be poorer in blacks, there are baseline differences in RV structure in function that vary by race/ethnicity in health, and we have shown a strong relationship between echocardiographic PH and heart failure admissions among black participants from the Jackson Heart Study [9, 49–51]. It is somewhat surprising then that women had improved survival compared to men given the baseline differences in race, although the models for both hemodynamics and survival accounted for race in the PH and precapillary PH groups.

Study limitations include those inherent to a retrospective cross-sectional study with data derived from electronic records. Still, excellent quality control is maintained in the CART database [20] and we undertook additional steps to ensure physiologic plausibility of all hemodynamic values assessed. There was a very small amount of missing data and at least one year of follow-up was required with confirmed survival status in the VA electronic health record and via outside claims data. Our study population was middle-aged US veterans with multiple medical comorbidities, roughly 50% of whom were inpatient during their right heart catheterizations, making “pure” WHO Group classification difficult and increasing the likelihood of confounding by indication and selection bias. Detailed data on medication use, including use of targeted PAH therapies, was not available. We adjusted for many factors and comorbid conditions that varied by sex as well as inpatient status with persistence of our findings, but residual confounding could certainly still exist and explain our findings.

The cohorts were predominantly male (reflecting the population of all US veterans) and the subset with precapillary PH was modest (n = 1930, with n = 120 women), limiting our power to detect hemodynamic differences in this group. The hemodynamic differences observed between men and women were small, raising the question of clinical relevance. We do not have repeated hemodynamic measures over time or additional measures of RV function (e.g., echocardiography or magnetic resonance imaging), both of which would potentially lend greater insight to our cross-sectional observations. CART does represent to our knowledge the largest available database of hemodynamics worldwide with extensive complementary information available on patient demographics, anthropometrics, and all comorbid conditions relevant to the pulmonary circulation, however, and the sample size still dwarfs those available in WHO Group 1 PAH (a rarer condition with much smaller cohorts of hemodynamic information available).

In summary, our data demonstrate clinical, hemodynamic, and outcome differences by sex among a general population of US veterans with elevated pulmonary pressures. Women have improved survival compared to men with PH and precapillary PH despite higher PVR and PAPP, which is perhaps explained by better RV adaptation. This work should generate additional hypotheses about unique sex-based phenotypes in all forms of pulmonary vascular disease.

## Disclaimer

The views expressed are those of the authors alone and do not represent the views of the Veterans Affairs or other Federal government agencies.
